# Demonstration of Directly Nanoimprinted Silica–Titania Large-Size Vertical Grating Couplers for Multichannel Photonic Sensor Development

**DOI:** 10.3390/ma18122771

**Published:** 2025-06-12

**Authors:** Andrzej Kaźmierczak, Cuma Tyszkiewicz, Magdalena Zięba, Mateusz Słowikowski, Krystian Pavłov, Maciej Filipiak, Jarosław Suszek, Filip Włodarczyk, Maciej Sypek, Paweł Kielan, Jerzy Kalwas, Ryszard Piramidowicz, Paweł Karasiński

**Affiliations:** 1 Institute of Microelectronics and Optoelectronic, Warsaw University of Technology, ul. Koszykowa 75, 00-662 Warsaw, Poland; ryszard.piramidowicz@pw.edu.pl; 2 Optoelectronics Department, Silesian University of Technology, ul. Krzywoustego 2, 44-100 Gliwice, Poland; cuma.tyszkiewicz@polsl.pl (C.T.); magdalena.zieba@polsl.pl (M.Z.); pawel.karasinski@polsl.pl (P.K.); 3 Centre for Advanced Materials and Technology (CEZAMAT), Warsaw University of Technology, ul. Poleczki 19, 02-822 Warsaw, Poland; mateusz.slowikowski@pw.edu.pl (M.S.); krystian.pavlov@pw.edu.pl (K.P.); maciej.filipiak@pw.edu.pl (M.F.); 4 Faculty of Physics, Warsaw University of Technology, ul. Koszykowa 75, 00-662 Warsaw, Poland; jaroslaw.suszek@pw.edu.pl (J.S.); filip.wlodarczyk.dokt@pw.edu.pl (F.W.); maciej.sypek@pw.edu.pl (M.S.); 5 Mechatronics Department, Silesian University of Technology, ul. Akademicka 10A, 44-100 Gliwice, Poland; pawel.kielan@polsl.pl; 6 VIGO Photonics S.A., ul. Poznańska 129/133, 05-850 Ożarów Mazowiecki, Poland; jkalwas@vigo.com.pl

**Keywords:** vertical grating coupler, sol-gel, silica-titania waveguides, multichannel photonic sensors, on-chip optical signal distribution

## Abstract

The article discusses the design, fabrication, and experimental evaluation of a large-area vertical grating coupler (VGC) enabling simultaneous coupling of multiple input optical beams. The presented VCG was fabricated by direct nanoimprinting of a grating pattern in a non-hardened SiO_X_:TiO_Y_ waveguide (WG) film. The WG film was deposited on a glass substrate using a combination of the sol–gel method and the dip-coating technique. The fabrication process allowed precise control of the waveguide film thickness and refractive index, as well as the VGC geometry. The relevance of the process was proved by a demonstration of optical coupling of multiple quasi-parallel input beams via the VGC to the WG layer. To make this possible, a dedicated optical coupling system was designed, including a polymer microlens array and optical fiber array positioned in a V-groove. This opens promising perspectives on using the proposed structure for the fabrication of low-cost multichannel optical sensor chips, as highlighted in the article’s final section.

## 1. Introduction

**Figure 1:**
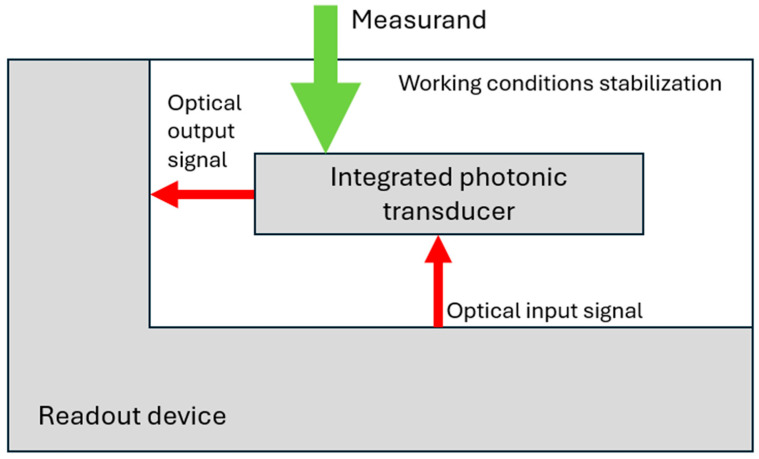
Schematic of sensor system with integrated photonic transducer.

Nowadays, integrated photonics development is dominated by its very mainstream application: optical telecommunication and data communication. Nonetheless, other emerging applications are being developed. Among the most promising are optical sensors [1], benefitting directly from a number of inherent features and offering the advantages of (i) high sensitivity, (ii) the possibility of operating with reduced volumes of sample and reagents, (iii) increased analysis speed, and (iv) the possibility of label-free operation, to name a few. In most of the proposed solutions, the device has the form of a system composed of a single-use disposable sensor chip and a designated readout device, as schematically pictured in Figure 1.

The readout device provides insertion and collection of the optical signal to/from the sensor chip and mechanical and environmental stabilization of the sensor chip. It also contains an interrogator unit allowing interpretation of the collected signals. In a minimal configuration, the sensor chip consists of a photonic layer including appropriately functionalized photonic transducers and microfluidic circuitry responsible for sample and reagent delivery. The sensor chip is commonly packaged in a cartridge, easing sensor holding and operation. The design of a sensor chip is governed by two fundamental, mutually contradictory considerations: high operation efficiency and low fabrication cost.

**Figure 2:**
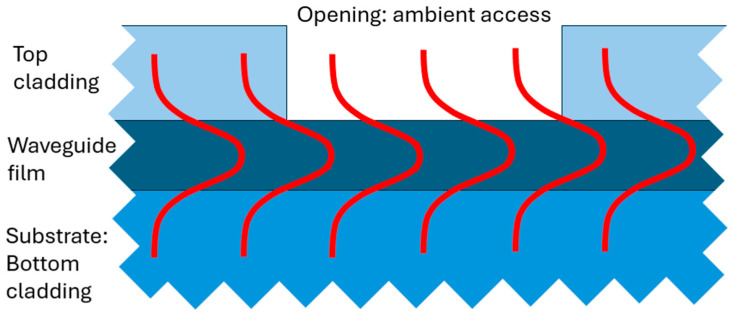
A schematic of the side view of a waveguide with a sensing opening in the top cladding layer, allowing the ambient environment to interact with a light signal guided by the waveguide.

Conventionally, the design of a photonic transducer is based on the incomplete confinement of the guided mode within the semiconductor or insulating waveguide core, resulting in partial light guiding in the waveguide cladding. When such a waveguide is partially stripped out of the top cladding, the opening allows the ambient environment to interfere with the propagated waveguide mode as schematically depicted in Figure 2.

**Figure 3:**
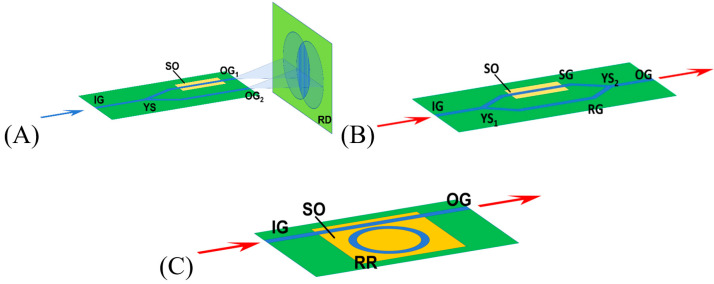
Schematic representations of exemplary layouts of integrated photonics devices for sensory applications: (**A**) Young interferometer; (**B**) Mach–Zehnder interferometer; (**C**) ring resonator.

In pursuit of the optimal solution, for sensing purposes, single-mode waveguides, having thickness and width close to the cut-off, have been proposed. These allow the evanescent field of the guided mode to expand even further from the waveguide core, and its propagation is therefore more affected by the ambient environment. To further enhance the sensitivity beyond the capabilities of the simple waveguide structure, resonant devices have been considered. Further, numerous integrated photonic sensor layouts have been proposed, including the Young [2,3] and Mach–Zehnder [4,5] interferometers, ring resonators [6,7,8,9], sub-wavelength gratings [10,11], and photonic crystal-based cavities [12,13]. Schematics of some of the above-mentioned integrated photonic sensor topologies are shown in Figure 3.

The following elements should be highlighted: the input waveguide (IG), waveguide splitter (YS), output waveguide (OG), sensor waveguide (SG), reference waveguide (RG), ring resonator (RR), and sensor opening (SO). It can be observed that, in addition to the fragment that is responsible for sensing (located in SO), more waveguide structures are necessary in the device layout.

All of the layouts presented in Figure 3 exploit the conventional approach, where the photonic chip is divided into three sections: (i) the input section, (ii) the photonic transducer section, and (iii) the output section. An emblematic case of such a configuration is shown in [14], where the photonic structure comprises an input grating coupler followed by a ring-resonator-based transducer and output grating coupler.

In laboratory routines, detecting multiple parameters (e.g., pathogens or pollutants) in the analyzed sample is frequently necessary, which leads to the need for multiparameter sensor development. Such a multiparameter sensor can be achieved by placing a number of photonics transducers on the sensor chip. Consequently, proper photonic circuitry must be developed to deliver and collect optical signals to/from multiple transducers. Two exemplary concepts for multichannel photonic sensors include (i) using a multitude of input structures, each delivering an optical signal to the particular transducer [15,16], or (ii) a single-input photonic structure followed by the optical distribution circuit [17] delivering the optical signal to each of the transducers.

The case of a multiparameter sensor system, in particular, illustrates the complexity of the photonic circuitry that needs to be used to construct the sensor chip, contrary to the idea of a low-cost and disposable device.

In the search for a more economical solution, it is worth investigating the possibility of developing simpler and easier-to-fabricate photonic circuitry while still maintaining the functionality of multichannel photonic sensors. In the present paper, we discuss the idea of using a single, large-area vertical grating coupler (VGC) acting both as a coupling device and as a multichannel transducer. The discussed VGC transducer is directly nanoimprinted onto an optimized SiO_X_:TiO_Y_ waveguide film and allows multichannel transmission, exhibiting significant potential to become a backbone of the prospective construction of multichannel photonic sensors.

## 2. Low-Cost Sol–Gel-Derived SiO_X_:TiO_Y_ Waveguides for Sensing Applications

Considering fabrication and the material technology point of view, it should be noted that many photonic integrated circuit (PIC) technologies have been used for integrated photonics sensor fabrication, including two of the mainstream ones, silicon-on-insulator (SOI) [18] and silicon nitride (SiN) [19], as well as numerous emerging technologies, e.g., those based on polymers [20] or composite materials (e.g., silica–titania). It is clear that the very mainstream PIC technology based on indium phosphide (InP) [21] waveguides is suitable for telecommunication applications rather than for the fabrication of photonic transducers.

One of the emerging technologies for integrated photonics sensor structure fabrication is based on silica–titania (SiO_X_:TiO_Y_) sol–gel-derived waveguide films deposited using a dip-coating technique. The advantages of this technology have been discussed in a number of research papers, including [22]. The most important of these advantages are as follows:

- The replacement of vapor-phase deposition techniques (e.g., CVD), where component ratios are adjusted by gas flow control, with liquid-phase deposition, where the components can be weighed;
- Precise adjustment of the thickness and refractive index (with the withdrawal speed from the sol and stoichiometric proportions of the SiO_2_ and TiO_2_ components in the sol);
- High chemical resistivity (low chance of oxidation as the material already consists of oxides);
- The possibility of direct nanoimprinting of the waveguide pattern, replacing two expensive technological steps (deep UV or electron-beam lithography and plasma etching are unavoidable in classical photonics technologies).

**Figure 4:**
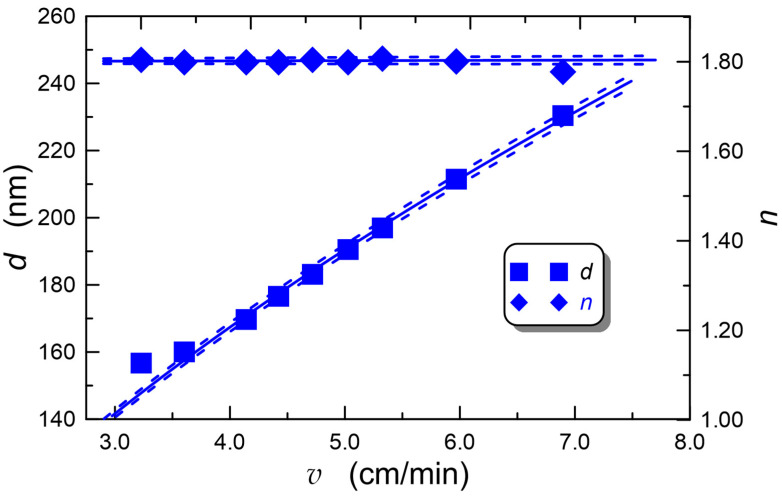
Technological characteristics of SiO_X_:TiO_Y_ WG film thickness and refractive index as function of substrate withdrawal speed from sol, demonstrating possibility of fine-tuning of SiO_X_:TiO_Y_ WG film properties.

Extensive studies of this technology have been conducted by a group from the Silesian University of Technology, leading to further technological development and maturation. Some exemplary experimental results showing the possibility of waveguide film thickness and refractive index control are shown in Figure 4.

As shown in Figure 4, it is possible to precisely control the WG film thickness by properly adjusting the withdrawal speed of the substrate from the sol. The experimental dependence *d* = *d*(*v*) was approximated by the function *d* = *Aξvα* + B, where a = 0.5740 ± 0.0018, *A* = (76.3 ± 0.2) nm, and *B* = (−1.69 ± 0.49) nm. Additionally, the experimental dependence of the refractive index *n* on the withdrawal speed was approximated by the linear function *n* = *av* + *n*_0_, obtaining *n*_0_ = 1.7947 ± 0.0012 and *a* = (0.000531 ± 0.000254) min/cm. The mentioned process does not affect the film’s refractive index, which is controlled by adjusting the stoichiometric proportions of silica and titania compounds in the sol. This provides flexibility and precise adjustment of the WG film parameters, allowing for the fabrication of structures optimized for the required application, e.g., structures optimized to have maximum sensitivity to ambient refractive index changes.

## 3. Directly Imprinted Vertical Grating Coupler Transducers

**Figure 5:**
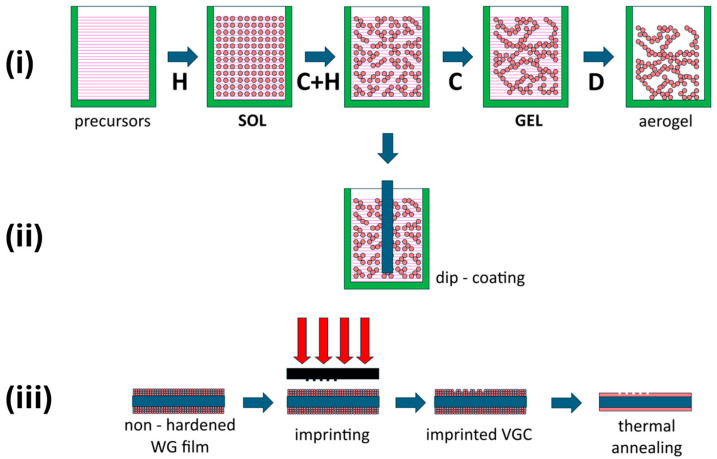
Schematic representation of fabrication process for SiO_X_:TiO_Y_ directly patterned VGCs: (**i**) sol formation and ageing process; (**ii**) dip-coating deposition; (**iii**) imprinting process. Acronyms: H—hydrolysis; C—condensation; D—drying.

The profitable feature of the above-described technology (unique when compared to mainstream PIC technologies) is the availability of direct nanoimprinting of photonic structure patterns on a WG film that has not yet been hardened in the thermal annealing process. This technique allows for a simplified fabrication process replacing two technological steps (i.e., e-beam or DUV lithography and plasma etching) with a single fabrication step. One photonic device that is especially well-suited for this fabrication technique is a shallowly patterned vertical grating coupler (VGC) that can act both as an optical in/out coupling device and as a photonic transducer for ambient refractive index sensing. The process flow for SiO_X_:TiO_Y_ directly patterned photonic structure fabrication is shown schematically in Figure 5.

The fabrication process of a VGC in a SiO_X_:TiO_Y_ WG film, as performed in this study, consisted of several steps. In the first step, a sol was synthesized from precursors to SiO_X_ and TiO_Y_. For this purpose, tetraethoxysilane (TEOS) and titanium (IV) ethoxide (TET) were used. In addition, the sol synthesis required deionized water, anhydrous ethanol (EtOH), and hydrochloric acid (HCl). The hydrolysis of the precursors was carried out separately. Then, after the hydrolysis of the precursors was completed, their solutions were mixed. The sol that was used to fabricate the WG films was characterized by a TEOS/TET molar ratio of 1:1 for the silica and titania precursors and a (TEOS + TET)/EtOH/H_2_0/HCl molar ratio of 1:7.23:6.97:0.32. In the next step, sol layers were deposited on clean glass substrates using the dip-coating process. The cleaning procedure involved mechanical washing in deionized water with added TRILUX universal detergent (from Analab, Gliwice, Poland), soaking in a solution of isopropyl alcohol, rinsing in deionized water, rinsing in acetone, and drying. Standard microscopic glass slides (with dimensions of 76 × 26 × 1 mm^3^) made of SCHOTT BK7 glass (from Pracownia Optyki Instrumentalnej, Warsaw, Poland) or Menzel-Glaser soda–lime glass (from Thermo Scientific, distributed by the company Bionovo, Legnica, Poland) were used. Immediately after the deposition of a sol layer, a VGC was formed by imprinting the pattern engraved in a silicon matrix. Finally, the structures were annealed at a temperature of 500 °C for 60 min. The final thickness of the fabricated WG films varied in the range from ~155 nm to ~230 nm, depending on the value of the substrate withdrawal speed, *v*.

Two technological setups were used for the implementation of the above-described process. The first setup was used for the deposition of the sol–gel-derived WG film with a dip-coating method. The setup included a vertical movement system (for substrate withdrawal from the sol) driven by a stepper motor and a gear belt drive system. The second setup (for direct nanoimprinting) included a cantilever-based system for the application of a precisely adjusted force on the processed sample. Both systems were custom-designed for the Department of Optoelectronics and are not commercially available. For the imprinted WG films, annealing in a WOMACH muff furnace controlled by a Shimaden SR93 (from Shimaden Co., Ltd., Tokyo, Japan) PID regulator was used.

The mechanism of coupling of a light beam, propagating in free space, to SiO_X_:TiO_Y_ WG films as a result of the diffraction of this beam on the VGC is discussed in [23]. The reader will find in that paper a detailed discussion of the relationship between the effective refractive index *n_eff_* of the WG film’s guided modes and the resonant coupling angle *φ_r_*. The work also discusses the resonant coupling angle’s sensitivity to changes in the refractive index of the ambient environment with respect to the thickness and refractive index of the WG film and VGC period *Λ*. One of the features of the developed VGCs is a detection limit as low as 5 × 10^−6^ RIU.

## 4. Large-Area VGCs for Multichannel Operation

The unquestionable advantage of the discussed SiO_X_-TiO_Y_ technology is that, to produce a fully operational photonic integrated circuit, the fabrication steps are limited to the deposition of a waveguiding film by the sol–gel method and the direct imprinting of the vertical grating coupler structure. Further, it must be emphasized that the VGC pattern on the slab waveguide film is the only pattern that should be fabricated to have a fully functional photonic sensor device. This contrasts with the numerous components in classical photonic sensor devices fabricated using mainstream PIC technology.

**Figure 6:**
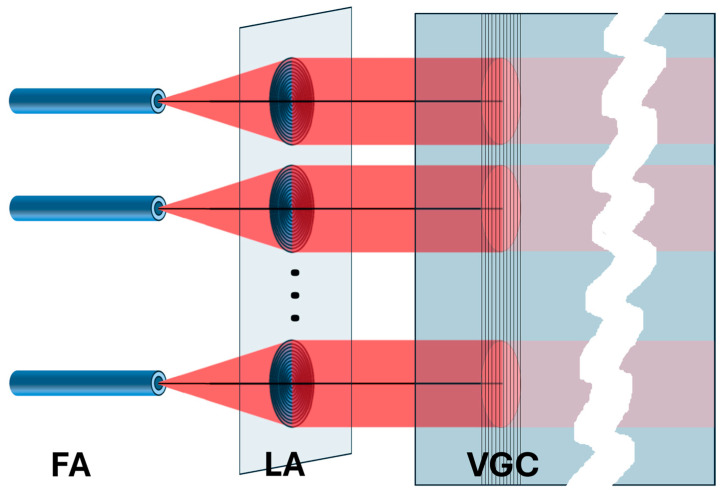
A schematic of the multichannel coupling scheme via a single large-area VGC. FA—optical fiber array; LA—microlens array; VGC—vertical grating coupler imprinted on SiO_X_:TiO_Y_ waveguide film.

This is a consequence of the fact that to obtain an almost non-diverging beam propagating in a waveguiding film after coupling through the VGC, it is enough to insert the quasi-parallel light beam into the VGC at the resonant incidence angle. A simple solution of such a system was presented in [24], including a single submillimeter-diameter polymer lens and a small-size VGC imprinted on a silica–titania WG film sufficient to accommodate a single optical path. This solution was a starting point for the analyses presented in this article. A consequence of quasi-parallel beam propagation in a slab waveguide is the possibility of multichannel operation of a VGC with a grating wide enough to accommodate a multitude of separated input quasi-parallel beams. This should occur when several beams (having propagation axes parallel to each other) fall on a large-area VGC at different locations at an optimal coupling angle. Further, these beams are coupled to the WG layer and then propagate to the end facet of the chip. This coupling is expected to be observed in the form of several parallel streaks of light on a photonic chip surface and several spots on the end facet. The implementation of multiple-beam propagation and coupling using a single VGC is schematically shown in Figure 6.

As one can see, several separated, quasi-parallel optical beams are propagated. As there is no reason for the beams to interfere with one another, a multichannel device could be obtained in this way. Each optical path, including the optical fiber, microlens, illuminated fragment of the VGC area, and subsequent fragment of the WG film area, where the light streak is located, can be considered an independent optical system. When particular fragments of the VGC are functionalized to selectively capture different molecules/substances/antigens, a multichannel sensor system can be created with functionalities similar to those of systems based on complex photonic distribution circuits or equipped with multiple input devices, even though the fabrication of the photonic chip is limited to WG film deposition and VGC patterning.

## 5. Instrumentation for Multichannel Optical Signal Coupling Scheme for Large-Area VGCs: Optical Fibers and Microlenses

**Figure 7:**
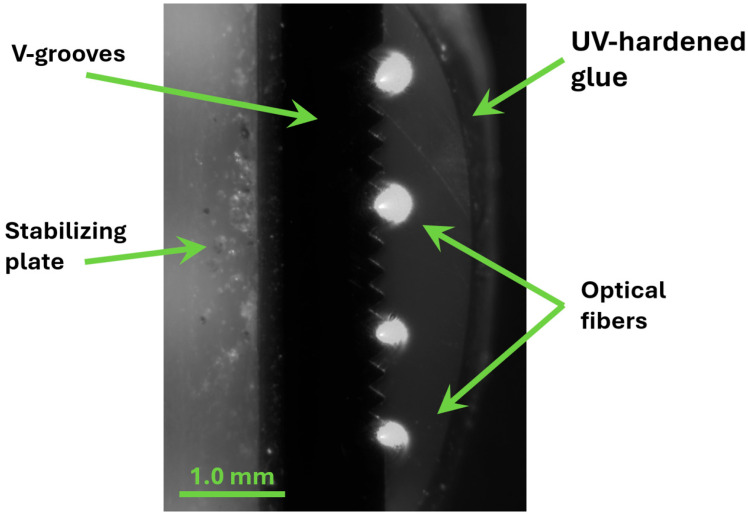
A photograph of the optical fiber transmission head composed of four single-mode optical fibers attached to an array of V-grooves.

To implement and demonstrate the coupling scheme depicted in Figure 6, a set of quasi-point sources emitting light in the same direction is needed, with a set of lenses having an appropriate focal length and geometry aligned adequately with the point sources. In our case, the optical signal was delivered via four polarization-maintaining (PM) single-mode optical fibers optimized for operation at wavelengths corresponding to the red part of the spectrum. The fibers were attached to a V-groove array. The exploited array had a pitch of 250 µm. The positions of the fiber cores in the V-grooves were measured with sub-micrometer precision to identify the exact fiber positions to which lenses in the lens array were adjusted. A photograph of the assembled optical fiber array in a V-groove is shown in Figure 7. For better visibility of the fibers, the photograph was taken with the fibers’ outputs illuminated by the propagating optical signals.

The complementary part of the optical path in the developed system was the lens array with parameters adjusted to the array of optical fibers. An example of a pluggable solution for multichannel fiber-to-chip coupling based on a lens array and a fiber array was discussed in [25] for PICs in SOI technology.

**Figure 8:**
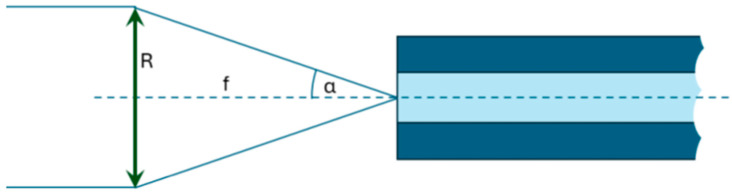
A schematic representation of the geometrical arrangement of the optical fiber and the lens in the system, showing the dependence between the lens and fiber parameters.

The implementation work began by determining the physical parameters of the optical system under study. The considered circumstances were the working wavelength of the system (λ) set to λ = 635 nm and the parameters of the optical fibers in the fiber array. The chosen fibers had a numerical aperture (NA) equal to 0.12. Consequently, half the acceptance angle was α = 6.89°. Taking into account that the simplest way to produce a quasi-parallel beam is to put a point source in a focal point of the lens (as shown in Figure 8), the NA of the fiber translated into the following desired relationship between the radius of the lens (*R*) and its focal length (*f*):

(1) $$\frac{R}{f}=tg\left(\alpha \right)=0.121$$

In the experiment, lenses of diameter *D* = 400 µm were used.

The next step was to design kinoform lenses that would be reproducible in polymer material using grayscale electron-beam lithography. This maskless technique enables fast prototyping of devices with different geometries, which is helpful for efficient optimization. The exposure was performed by mapping and assigning individual height and dose values for each patterned pixel (doses in the range of 0–255). The height of each (*x*,*y*) point of the kinoform lens of diameter *D* and radius of curvature *R* was calculated using the following equation:

(2) $$h\left(x,y\right)=mod\left[max\left(\sqrt{R^{2}-x^{2}-y^{2}}-\sqrt{R^{2}-\frac{D^{2}}{4}},0\right),h_{max}-h_{min}\right]$$

A kinoform lens is divided into zones. To achieve low phase mismatch between light beams passing through subsequent zones, the optical path difference introduced by every zone should be a multiple of the illumination wavelength. The lenses were designed to operate with a light source at wavelength *λ* = 633 nm. Assuming that the refractive index of PMMA at this wavelength is *n* = 1.48, the kinoform height can be obtained using the equation below:

(3) $$h_{max}-h_{min}=\frac{\lambda }{n-1}=\frac{633\;nm}{1.48-1}\approx 1319\;nm$$

**Figure 9:**
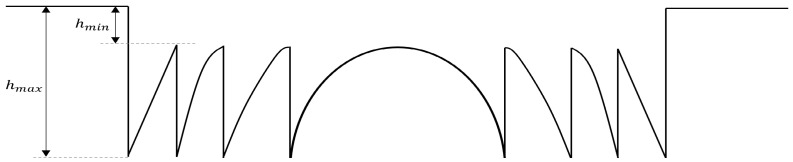
A schematic of the kinoform lens profile used in the lens design; *h_min_* and *h_max_* stand for the minimum and maximum depths of the kinoform shape.

Figure 9 shows a schematic of the designed kinoform lens cross-section.

The lenses were designed to be fabricated in a 2.65 µm thick polymethyl methacrylate (PMMA) layer. The minimum (*h_min_*) and maximum (*h_max_*) depths of the kinoform shape were determined based on performed simulations and the contrast curve of the material in use. The resulting parameters were *h_min_* = 200 nm and *h_max_* = 1519 nm.

The lenses’ layouts were subsequently loaded into GenIsys BEAMER v5.5.2 software [26], and a map was prepared for electron-beam lithography based on the contrast curve used earlier. A base dose of 1000 µC/cm^2^ was used, and the pixel size was set to 0.96 µm. For the maximum exposure depth (*h_max_*), 0.438 and 0.1413 dose multipliers were set as the *h_max_* and *h_min_* doses, respectively.

**Figure 10:**
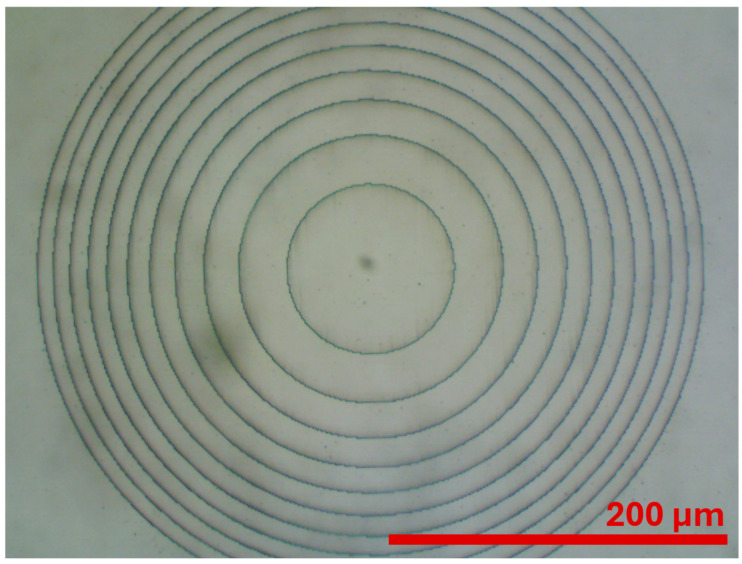
An optical microscope image of a PMMA kinoform lens fabricated at CEZAMAT from the lens array used in the experiments.

The final step was to prepare the lens array. Four lenses were arranged in a line at different intervals, corresponding to the distances between optical fibers in a V-groove. An optical microscope image of a fabricated lens is shown in Figure 10.

## 6. Design and Fabrication of the Large-Area Silica–Titania VGC

**Figure 11:**
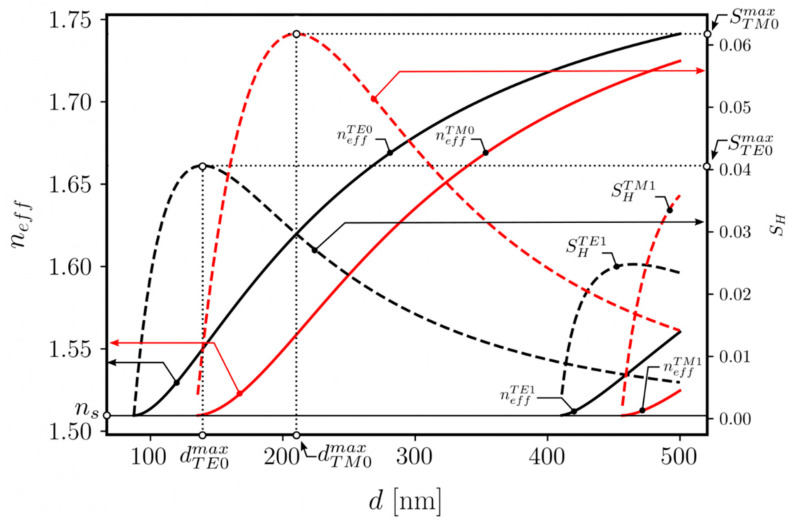
Characteristics showing dependence of WG film’s guided-mode effective index *n_eff_* and homogeneous sensitivity *S_H_* on WG film thickness *d* for WG film refractive index *n_f_* = 1.81.

WG films with large-size VGCs were designed and fabricated to demonstrate multichannel operation. The structures had the following parameters: the WG film refractive index and thickness were *n_f_* = 1.81 and *d*= 218 nm, respectively, and the grating period was *Λ* = 417 nm. This particular WG film thickness was selected because, for this value, the sensitivity of the TM_0_ mode is at a maximum. The theoretical analysis is displayed in Figure 11, with the characteristics showing variations in the effective refractive index *n_eff_* and homogeneous sensitivity *S_H_* of the WG film’s guided modes. The characteristics of *S_H_*(*d*) were calculated by analyzing the modal characteristics of *n_eff_*(*d*). Homogeneous sensitivity is defined by the following equation [27]:

(4) $$S_{H}={\left.\frac{{dn}_{eff}}{{dn}_{c}}\right|}_{d}$$

The derivative in (4) was approximated by a difference quotient, assuming that the increase in the cover refractive index was Δ*n_c_* = 0.0001.

**Figure 12:**
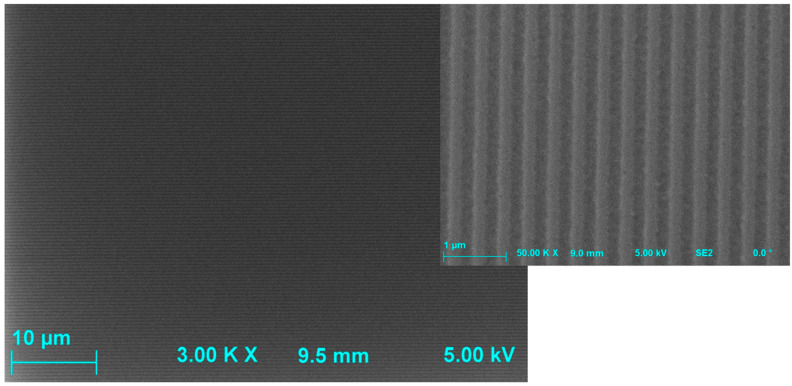
SEM images of the fabricated VGC.

A larger area and a close-up SEM image of the fabricated VGC structure are shown in Figure 12.

**Figure 13:**
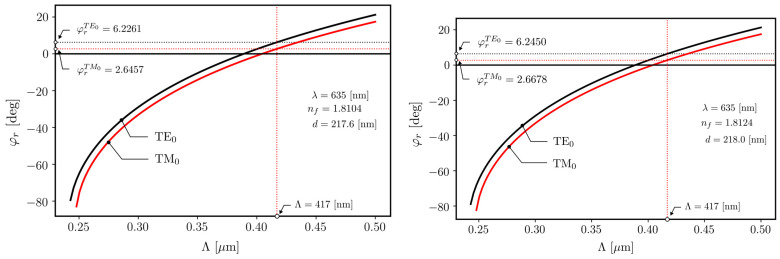
Simulations of the resonant coupling angle vs. the VGC period for both fundamental modes and two pairs of measured values for the WG film’s thickness and refractive index.

The VGC was imprinted on non-hardened silica–titania film deposited on a BK7 glass substrate of length 76 mm and width 26 mm. The imprinted VGC had a width equal to the substrate’s width. The WG film’s thickness *d* and refractive index *n_f_* were measured on a line parallel to the VGC at two points. The first one lay in the center of the substrate, and the second was approximately 5 mm closer to the substrate’s long edge. The results of those measurements are given next to the lines on two characteristics presented in Figure 13. They are only slightly different. This indicates that the WG layer thickness was uniform in the direction of the VGC. The characteristics present a variation in the in-coupling angle of both fundamental modes with the grating coupler’s period *Λ*. One can see that both fundamental modes are resonantly excited for positive angles *φ_r_* by diffraction in the first order on the VGC. The thickness and refractive index of the WG film were measured using a SENTECH SE400adv monochromatic ellipsometer (from Sentech Instruments GmbH, Berlin, Germany).

## 7. Demonstration of Multichannel Coupling to Large-Area VGC

**Figure 14:**
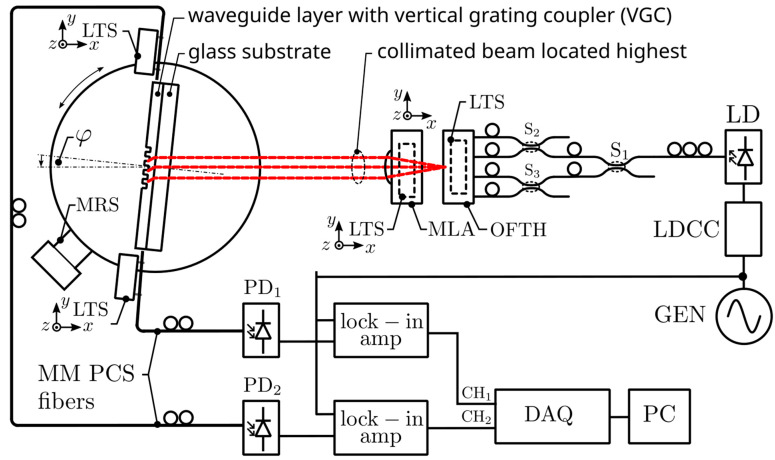
Schematics of the experimental setup for multichannel optical signal coupling to the VGC. DAQ—data acquisition card; GEN—sine wave generator; LD—laser diode; LDCC—laser diode current controller; LTS—linear translational stage; MLA—array of microlenses; MM PCS—multimode plastic-clad silica; MRS—motorized rotational stage; OFTH—optical fiber transmission head; PC—personal computer; PD_1_, PD_2_—photodiodes; S_1_, S_2_, S_3_—single-mode fiber optic splitters.

The ultimate goal of this work was experimental confirmation of the availability of multichannel optical signal transmission through a single large-area VGC. The measurement setup shown in Figure 14 was assembled to provide such experimental proof.

The photonic chip was placed in a dedicated holder mounted on a Thorlabs NR360S (from Thorlabs Inc., Newton, NJ, USA) motorized 360° rotation stage. The chip was oriented so that the light beams excited in the WG film propagated parallel to the chip’s long edge and were received at both opposite chip facets by two multimode PCS fibers having cores of diameter 250 µm. The PCS optical fibers were mounted on linear translational stages, which allowed for fine-tuning of the position of incident beams on the VGC. The light intensity of optical signals received from opposite facets of the chip was converted to an electric voltage by two PDA36A2 silicon photodiodes supported with FEMTO LIA-MV-150 lock-in amplifiers (from Femto Messtechnik GmbH, Berlin, Germany). The characteristics of luminous intensity as a function of the angle of incidence φ were recorded by an NI-6009 data acquisition card (from National Instruments, Austin, TX, USA). The light source in the presented measuring system was a single-mode pigtailed laser diode operating at wavelength λ = 635 nm. Among those at our disposal, this laser operated at the wavelength closest to 633 nm. The light beam generated by the laser was evenly distributed among four single-mode optical fibers, the ends of which were mounted in the optical fiber transmission head (OFTH). The latter is presented in Figure 7. Divergent beams of light emitted from the OFTH were collimated by an array of microlenses (MLA). Both the OFTH and MLA were mounted on linear translation stages. A number of experiments were conducted to verify the proper operation of the designed optical signal distribution system. In the first experiment, for simplicity, the optical signal was delivered via only two optical fibers in a V-groove. The position of the lens array was adjusted appropriately to produce two quasi-parallel beams that illuminated the VGC of the optical chip placed in a holder attached to the rotation stage. The coupling angle was adjusted to optimize the output signal. Figure 13 demonstrates a photograph of a photonic chip mounted in the holder, illuminated at the VGC at the optimal (resonant) coupling angle with two beams separated by approximately 2500 µm.

**Figure 15:**
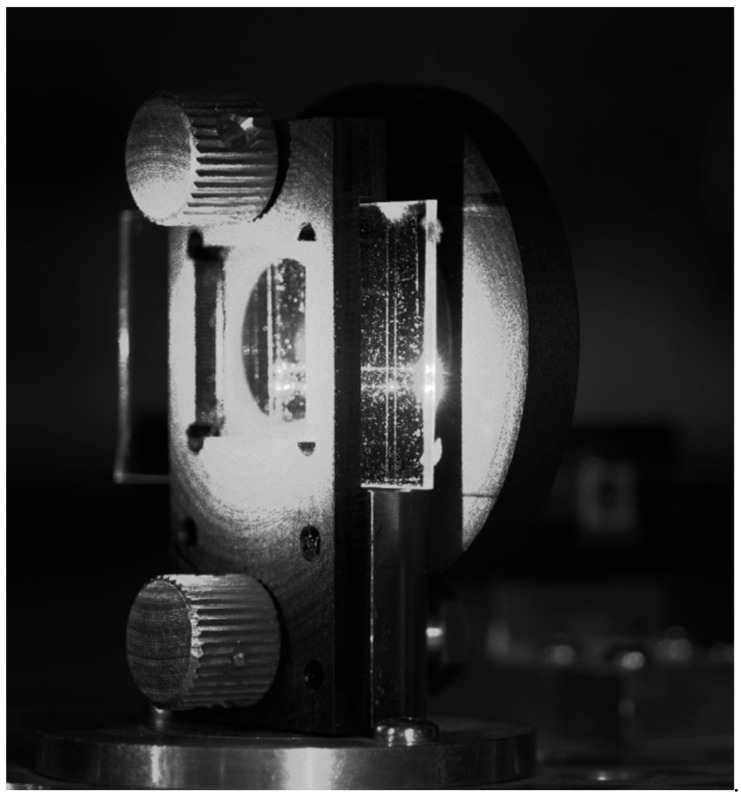
An image of the photonic chip in a chip holder illuminated with two quasi-parallel beams at the optimal coupling angle.

The image in Figure 15 shows the chip from the side of its substrate, which was illuminated by the beams emitted by the OFTH and collimated by the MLA. The VGC was on the opposite side of the chip. Notably, a fraction of the optical signal was transmitted outside the lens circumference. As a result, one can observe a light spot on the chip holder.

**Figure 16:**
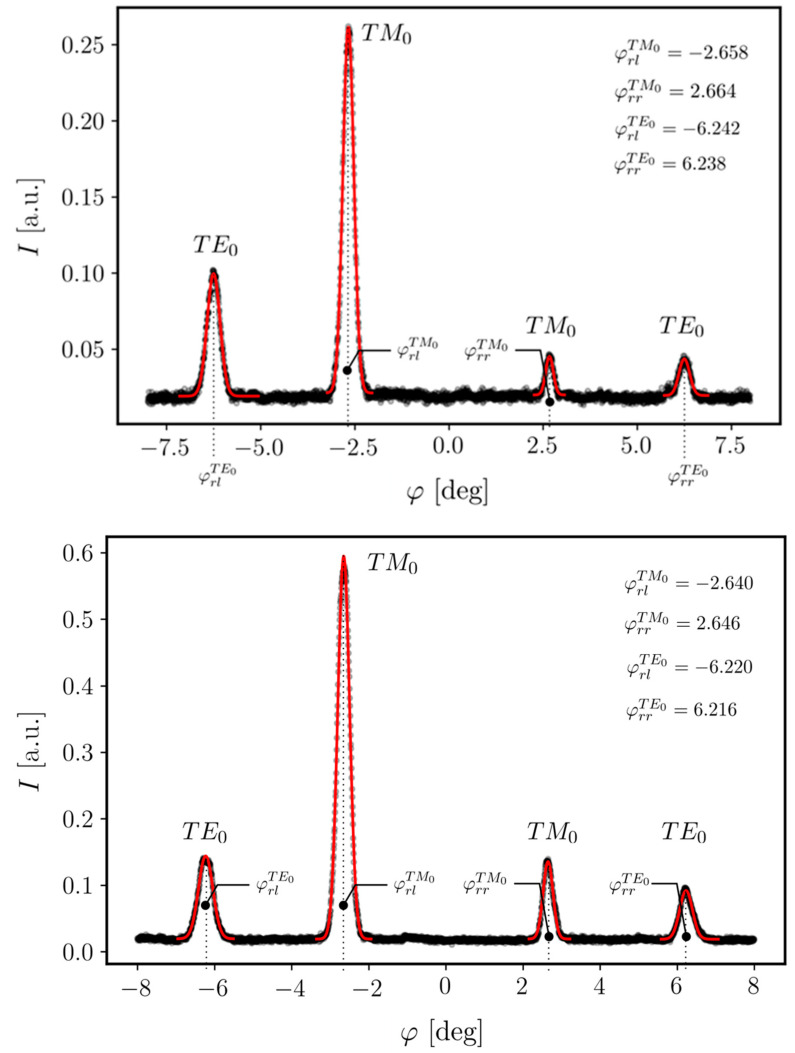
Angle transmission spectra collected at the output facets of the photonic chip for upper and lower light streaks (Figure 13).

One can also observe two bright streaks, originating at the VGC and reaching the chip end facet, with two even brighter output spots. As already mentioned, to collect the angular characteristics of light intensity coupled into the WG film, a pair of PCS optical fibers were placed near the end facets of the chip on both sides of the VGC. Depending on the orientation of the collimated beam with respect to the normal to the chip, the modes can be excited toward the left-hand (negative *φ*) or right-hand side (positive *φ*) of the chip. Each coupled beam was measured separately because the position of the detection PCS fibers at the chip’s edges had to be changed. The coupling characteristics (i.e., the optical signal intensity vs. the coupling angle) of the lower and upper beams are presented in Figure 16.

Four resonant coupling angles can be distinguished for both optical paths (i.e., for both light streaks). The resonant coupling peaks are symmetrically distributed on both sides with respect to the normal incidence angle. Two of them, located closer to the normal, correspond to the optical coupling of the TM-polarized optical signal, while the two more distant peaks correspond to the TE-polarized signal coupling. It is notable that the obtained experimental results are very close to the simulation results shown in Figure 11. The slight deviation between the spectra collected from the output spots of two streaks may originate from slight differences in the WG film geometry along the grating width or from slight variations in the VGC relief itself.

**Figure 17:**
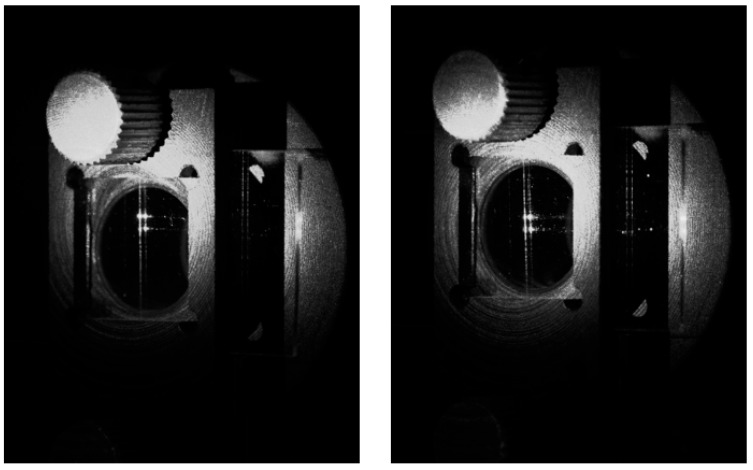
Images of the photonic chip illuminated at different positions on the VGC at the optimal coupling angle.

After the optical signal coupling was confirmed, the VGC position was adjusted in height without affecting other settings of the optical path to verify the coupling availability of the VGC along its entire length. Images presenting two collimated beams coupled to the middle and upper sections of the same VGC are shown in Figure 17.

As one can easily observe, the optical signal can be coupled through the VGC at different positions along the VGC’s width. Therefore, the opinion that the VGC is operational along its entire width is fully justified. This confirms the suitability of constructing an optical system with many optical channels using such a VGC.

**Figure 18:**
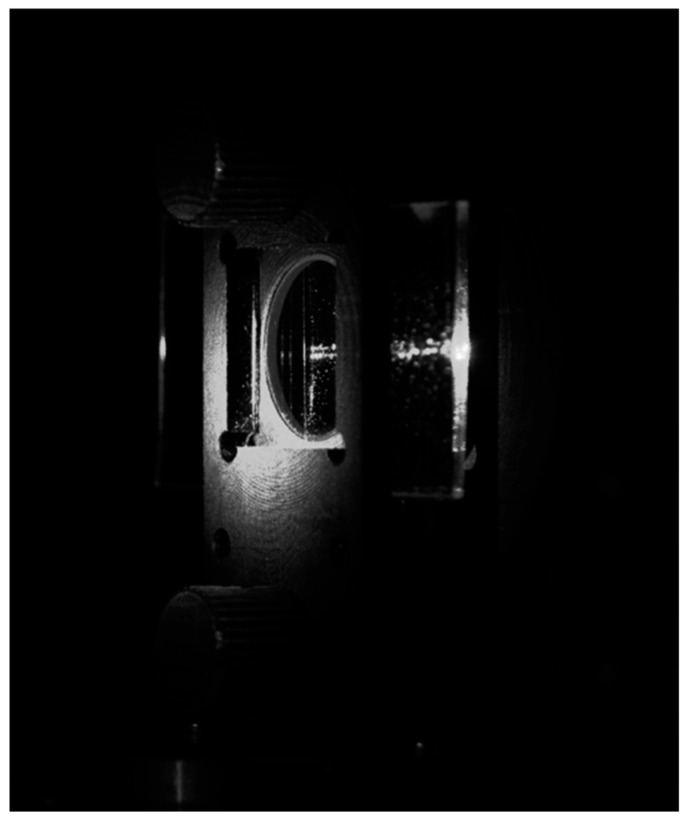
An image of the photonic chip illuminated at the optimal coupling angle with two optical beams separated by 1 mm.

The system’s operation was further verified by coupling two optical beams separated by 1 mm, as shown in Figure 18.

It is noteworthy that the beams with centers separated by 1000 µm are well-separated and do not interfere with one another; no signal crosstalk between these beams can be observed. This enables locating at least 25 sensor fields on a 1-inch-wide VGC.

**Figure 19:**
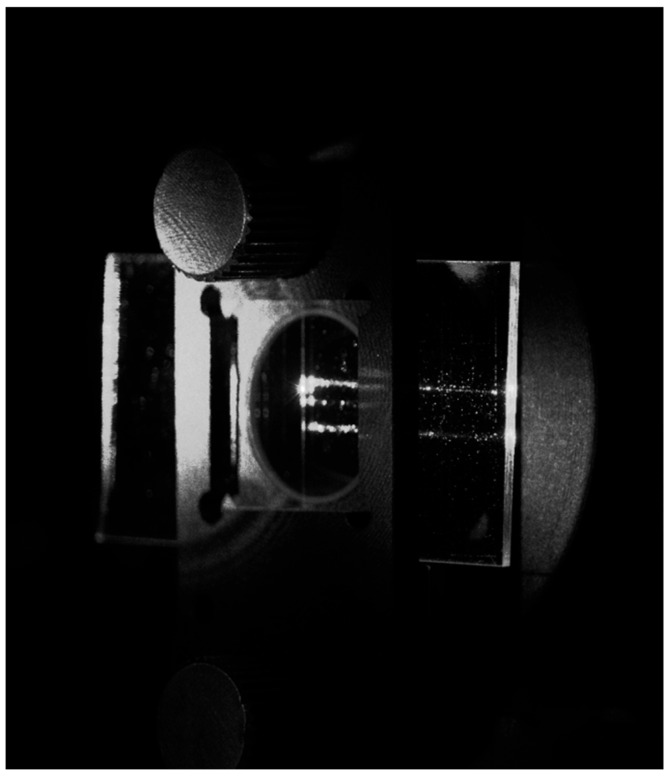
An image of the photonic chip illuminated at the optimal coupling angle with three optical beams.

The possibility of coupling a multitude of signals with the proposed system was further demonstrated with the simultaneous coupling of three optical beams to the VGC, as shown in Figure 19.

As one can see, three optical beams were coupled to the VGC, two of them producing light streaks reaching the end facet of the chip. However, the middle streak did not reach the end facet. It should be stressed that the optical coupling of three beams requires almost perfect alignment between three optical fibers in a V-groove and three lenses in a lens array. It can be seen in the figure that some misalignments appeared, forbidding ideal signal coupling for all three beams.

## 8. Discussion and Conclusions

In this article, successful fabrication of a large-area VGC for multichannel sensor construction was demonstrated. Notably, the VGC was fabricated through low-cost direct nanoimprinting on low-cost sol–gel-derived SiO_X_:TiO_Y_ WG films. Optical signal coupling to almost the entire width of the VGC was demonstrated. The optimal optical coupling angles were almost identical to those predicted by simulation, showing proper control of the fabrication process. Proper coupling of two optical beams with beam centers separated by 2.5 mm and by 1.0 mm was demonstrated, proving the correctness of the proposed optical signal coupling scheme based on an array of optical fibers and an array of corresponding polymer kinoform microlenses. Optical signal coupling to the VGC from more beams was not as good as two-beam coupling, most probably due to imperfect alignment between the optical fibers and the lens array. This result motivates further development of the system, including better identification of the optical fibers’ position in a V-groove, leading to the possibility of simultaneous coupling of many signals to the same VGC. Further work will also include the development of even more channel systems, as well as the integration of microfluidic devices with the photonic chip.

## Data Availability

The original contributions presented in this study are included in the article. Further inquiries can be directed to the corresponding author.
